# Cryptococcal Osteomyelitis in a Virally Suppressed Adolescent Living With HIV: A Case Report

**DOI:** 10.1155/crdi/4804916

**Published:** 2026-05-25

**Authors:** Linda A. Mandikiyana Chirimuta, Christina Rambanapasi, David Musorowegomo, Gilbert Nyatanga, Augustine Mapfumo, Eliot F. Chikati

**Affiliations:** ^1^ Pediatric Department, Newlands Clinic, Harare, Zimbabwe, newlandsclinic.org.zw; ^2^ Department of Pediatrics, Sally Mugabe Children’s Hospital, Harare, Zimbabwe; ^3^ Department of Orthopedics, Faculty of Medicine and Health Sciences, Midlands State University, Gweru, Zimbabwe, msu.ac.zw; ^4^ Department of Orthopedics, Sally Mugabe Central Hospital, Harare, Zimbabwe; ^5^ Department of Orthopedics and Trauma, College of Surgeons of East, Central and Southern Africa, Arusha, Tanzania

**Keywords:** amphotericin B, antifungal treatment, case report, *Cryptococcus neoformans*, fluconazole, HIV infection, osteomyelitis

## Abstract

*Cryptococcal neoformans* infection of the central nervous system (CNS) is well‐documented in Sub‐Saharan Africa due to its association with the human immunodeficiency virus (HIV) pandemic. While cryptococcal infections can occur at sites other than the CNS, such as in the bones, these instances are less common and have been inadequately documented in the region. Orthopedic cryptococcal infections arise when the organism is inhaled from the environment and subsequently disseminates from the lungs via the bloodstream to the bones. Due to its low incidence and atypical manifestations, cryptococcal osteomyelitis may be overlooked, leading to delayed treatment and potential complications. This case report describes a 12‐year‐old female patient who presented with pain and swelling in both knee joints one year after initiating treatment for cryptococcal meningitis, which had been only partially effective. On examination, both knee joints exhibited swelling and tenderness, and imaging revealed bilateral pathological fractures in the distal third of the femurs. Histological analysis demonstrated numerous fungal elements consistent with *Cryptococcus neoformans*. Treatment was initiated with liposomal amphotericin B at a single dose of 10 mg/kg, in conjunction with flucytosine at 100 mg/kg/day and fluconazole at 1200 mg/day. This was followed by a consolidation phase with fluconazole at 800 mg/day, after which the patient was maintained on fluconazole at 200 mg/day. After 52 weeks of treatment, the patient exhibited a favorable clinical response, with successful union of the fractures. In individuals living with HIV, *Cryptococcus neoformans* may present atypically with involvement of sites outside the CNS, including the skeletal system. Although cryptococcal osteomyelitis is rare, it should be considered in the differential diagnosis in this population. Favorable outcomes can be achieved with prolonged antifungal therapy.

## 1. Introduction

*Cryptococcus neoformans* is an invasive fungal infection, particularly prevalent in Sub‐Saharan Africa due to its association with the human immunodeficiency virus (HIV) pandemic. This organism is opportunistic and affects individuals with defects in cell‐mediated immunity, such as people living with HIV (PLHIV) [1]. The organism is ubiquitous in the environment, commonly found in decaying soil matter, certain tree species, and avian excreta [2]. Cryptococcal infection begins when environmental basidiospores (small yeast cells measuring less than 5 μm) are inhaled and deposited into the pulmonary alveoli [3], before disseminating from the lungs to cause infections in various tissues, typically the central nervous system (CNS). However, other organs such as the skin, eye, prostate, and rarely, the skeletal system may be affected [4].

Orthopedic cryptococcosis is poorly documented in Sub‐Saharan Africa compared to the widely documented CNS cryptococcal infection. Most literature on orthopedic cryptococcosis originates from the West and is primarily from individuals without known immunosuppression. Orthopedic cryptococcal infections occur in less than 10% of patients with disseminated cryptococcosis [5], most frequently in the vertebrae and long bones, with the knee being the most commonly infected joint [6, 7]. Osteomyelitis is generally preceded by fungemia, but in some cases, it may arise from direct inoculation of the etiologic agent [1].

Due to its low incidence, atypical manifestations, and nonspecific imaging findings, cryptococcal osteomyelitis poses a diagnostic challenge and is easily misdiagnosed, delaying treatment in many cases [8]. Delays in diagnosis may result in complications such as pathological fractures.

We describe a case of cryptococcal osteomyelitis of the distal femurs bilaterally following incompletely treated cryptococcal meningitis in an adolescent from Zimbabwe.

## 2. Case Presentation

A 12‐year‐old female patient from Zimbabwe, living with HIV, was diagnosed at nine years old (Table 1 for timeline). She acquired HIV via mother‐to‐child transmission, and both her parents are living with HIV. She commenced antiretroviral treatment (ART) two years after diagnosis, in February 2021, and was prescribed abacavir (ABC)/lamivudine (3TC)/lopinavir/ritonavir (LPV/r). She was WHO Clinical stage II, and no baseline CD4 count or viral load was obtained.

**TABLE 1:** Timeline of clinical events.



Abbreviations: 3TC, lamivudine; ABC, abacavir; ART, antiretroviral treatment; CSF, cerebrospinal fluid; DTG, dolutegravir; HIV, human immunodeficiency virus; L‐AmB, liposomal amphotericin B; LPV/r, lopinavir/ritonavir; MRI, magnetic resonance imaging.

In February 2022, she was diagnosed with virological treatment failure and switched to ABC/3TC/dolutegravir (DTG). Two weeks later, she presented with a severe headache and vomiting. A diagnosis of cryptococcal meningitis immune reconstitution inflammatory syndrome (IRIS) was made after her cerebrospinal fluid (CSF) was India ink positive on microscopy and cryptococcal antigen (CrAG) positive. Her CD4 count at this time was 55 cells/μL. She was prescribed liposomal amphotericin B 10 mg/kg intravenously once, with flucytosine at 100 mg/kg/day and fluconazole at 1200 mg/day for 2 weeks. Unfortunately, fluconazole and flucytosine were poorly tolerated due to the pill burden and poor palatability of the tablets; therefore, an adherence assessment made using a pill count revealed that only half of the doses were taken. Due to the inadequate treatment and poor tolerance, the induction treatment was extended by another week, and fluconazole was reduced to 800 mg/day. A CSF fungal culture was not performed at the end of induction treatment. For consolidation, she was given fluconazole 600 mg/day for 8 weeks. ART was withheld for 4 weeks from the start of cryptococcal meningitis treatment before being recommenced.

Adherence to medicines remained poor during treatment, despite interventions. At Week 5 of ART reinitiation, the patient developed cryptococcal meningitis, confirmed by a CSF culture that grew *C. neoformans*. The patient received seven doses of liposomal amphotericin B at 5 mg/kg/dose. Administration was slow over several days due to challenges with side effects of amphotericin B infusion, namely, hypokalemia and hypomagnesemia, which would be corrected before continuing with treatment. Flucytosine 100 mg/kg/day and fluconazole 800 mg daily were given for 2 weeks. Two weeks postretreatment, a repeat CSF culture remained positive for *C. neoformans*, resistant to fluconazole but sensitive to flucytosine and voriconazole. Fluconazole was the only azole available and continued at 600 mg/day for 8 weeks, then 200 mg/day thereafter. ART and adherence support were continued.

**FIGURE 1:**
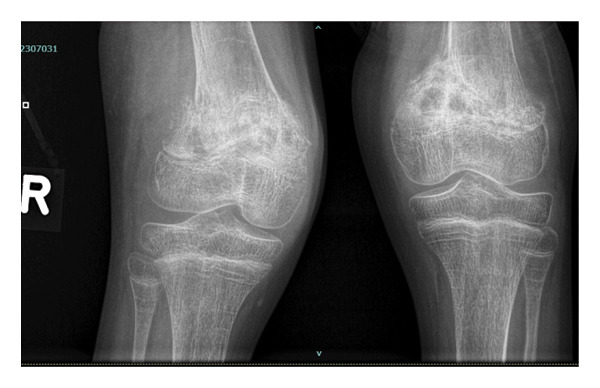
X‐Ray of knees showing bilateral Salter–Harris Type II fractures of the distal femur, with the right femur fracture displaced.

A year later, the patient developed painful, swollen knees for 3 months, initially the left knee, and then, 2 weeks later, the right knee. No other joints were swollen, and she had no constitutional symptoms nor a preceding history of trauma. Upon examination, both knees were swollen, warm, and tender with reduced range of movement. Joint aspiration was dry. Her viral load was < 20 copies/mL and CD4 count 267 cells/μL. Antinuclear antibodies and rheumatoid factor were negative. X‐Rays showed a mixed picture of sclerosis and lysis, cystic lesions, and bilateral Salter–Harris Type II fractures of the distal femur, with the right femur fracture displaced (Figure 1).

**FIGURE 2:**
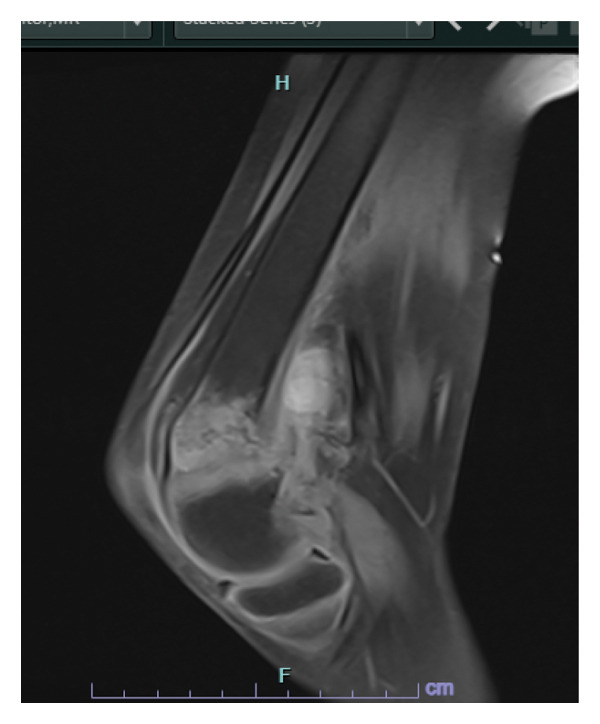
Magnetic resonance imaging of the right knee showed a transverse distal femoral fracture associated with periosteal elevation and intermediate callus formation.

Magnetic resonance imaging of the right knee confirmed a transverse distal femoral fracture associated with periosteal elevation and intermediate callus formation. Multiple enhancing popliteal fossa nodes were seen, with the largest measuring 0.6 cm. There was no associated knee joint effusion (Figure 2).

**FIGURE 3:**
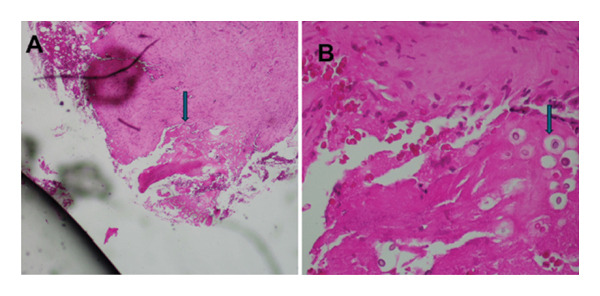
Histology slide from a bone biopsy taken from the patient, showing in (A), magnification 4× shows fibroconnective tissue infiltrated by small round to oval yeasts, in (B), magnification 20× close up of the yeast showing the thick capsule.

The patient was taken for surgical decompression of the distal femur, where approximately 150 mL of pus was drained, and a washout was performed. A bone biopsy was performed and sent for histology, microscopy, and culture. Postoperatively, intravenous ceftriaxone and cloxacillin were given for 2 weeks. Bacterial culture and *Mycobacterium tuberculosis* culture were negative. *Mycobacterium tuberculosis* polymerase chain reaction and alcohol acid‐fast bacilli tests were negative. Later, histology showed fragments of dead bone and fibroconnective tissue with extensive necrotizing epithelioid granulomas. Numerous fungal specimens were seen, with the morphological appearance of *Cryptococcus neoformans* (Figure 3). A diagnosis of cryptococcal osteomyelitis was made. There was an absence of small round blue cells on histology, ruling out Ewing sarcoma.

**FIGURE 4:**
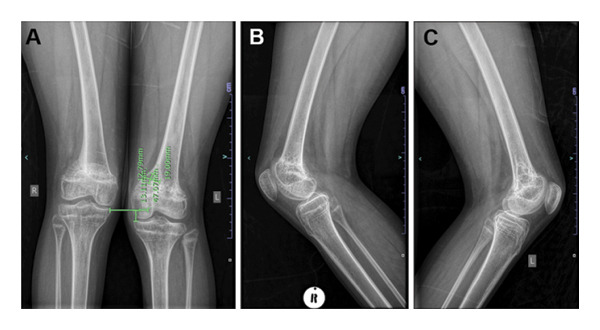
Repeat X‐rays taken after 52 weeks of antifungal treatment, showing in (A, C), persistence of the cystic change in the distal left femoral metaphysis. In (B), complete union of the pathological fracture of the distal right femur with almost complete fusion of the distal right femoral epiphysis.

Consequently, liposomal amphotericin B was administered at 4 mg/kg/day for 7 days, with flucytosine 100 mg/kg/day and fluconazole 800 mg/day for 2 weeks, followed by fluconazole 600 mg/day for 10 weeks and then fluconazole 200 mg/day thereafter. ART was continued without interruption. After 52 weeks of treatment, the patient showed significant clinical improvement. Repeat X‐rays indicated complete union of the pathological fracture of the distal right femur with almost complete fusion of the distal right femoral epiphysis. The cystic change in the distal left femoral metaphysis persisted (Figure 4).

## 3. Discussion

In Sub‐Saharan Africa, cryptococcosis is commonly known to cause meningitis or, less frequently, pulmonary disease in immunocompromised individuals with advanced HIV infection. Involvement of sites other than the CNS and lungs may occur, but has been poorly documented in this region.

To our knowledge, our case is one of the very few documented instances of cryptococcal osteomyelitis in a person living with HIV in Sub‐Saharan Africa and the first documented case in Zimbabwe. Our patient initially had cryptococcal meningitis, which was unfortunately incompletely treated due to suboptimal adherence to the prescribed therapy. She later developed bilateral cryptococcal osteomyelitis of the distal femurs, resulting in pathological fractures of the lower third of both tibias.

One limitation of this case report is that adherence assessment relied on pill counts and the patient’s self‐report, without objective measurement of drug levels. The disease onset of fungal osteomyelitis is typically subacute, with symptoms including local pain, tenderness, and swelling, sometimes accompanied by fever and weakness. Local destruction, such as fracture and deformity, can be the first presenting feature [8, 9]. Imaging findings usually comprise irregular osteolytic lesions with or without periosteal elevation. These X‐ray findings are nonspecific and may also be seen in conditions such as bacterial osteomyelitis, tuberculosis, and osteosarcoma.

Testing for CrAG in the serum may assist in diagnosis, although there are reports of cryptococcal bone and joint disease despite negative antigen tests [10]. Diagnosis is established by the isolation of the organism on culture from synovial fluid, tissue specimens, or draining sinus tracts. Histopathological examination of biopsy material demonstrates multinucleated giant cells and granuloma formation. Special stains applied to bone and synovial tissue, including methenamine silver and periodic acid–Schiff, can reveal the presence of characteristic budding cryptococci [8].

Studies of cryptococcal infection treatment at sites other than the lungs and CNS are scanty. Generally, infection at a single site without CNS disease, fungemia, or risk factors for immunosuppression may be managed with fluconazole 400 mg (6 mg/kg) once daily for 6–12 months [11]. In our case, due to the patient’s history of CNS disease and fluconazole resistance in past cultures, we initiated treatment with liposomal amphotericin B (5 mg/kg/day) for seven days, along with flucytosine (100 mg/kg/day) and fluconazole 800 mg daily for two weeks, followed by fluconazole 600 mg/day for 8 weeks and then 200 mg a day thereafter.

Another explanation for the minimal improvement in the osteomyelitis radiologically, after 2 months of treatment, was due to the biological nature of the *Cryptococcus* organism. Fungi attach to the bone surface and form a biofilm composed of fungi, glycoproteins, and extracellular material. This biofilm renders antifungal resistance and potentially forms a nidus of infection that can pose a risk of disseminated cryptococcosis in the future. Cryptococcal osteomyelitis with established biofilm should be cured by surgical excision of the infected bone. Management of chronic cryptococcal osteomyelitis should follow the following principles: surgical excision, temporal fracture stabilization, antifungal treatment, dead space management, soft tissue reconstruction, and definitive fixation. Our case is still awaiting surgical excision and dead space management.

Persistent or relapsed cryptococcosis may occur due to a continued immunocompromised state, inadequate fungal eradication with therapy, or resistance to treatment. Persistent infection is defined as continuously positive cultures after 4 weeks of therapy, while relapse requires positive cultures from a previously sterile site and new signs and symptoms at the original site of disease [12]. Our patient likely developed a persistent infection due to poor adherence to treatment following her initial cryptococcal meningitis. Management of persistent and relapsed infections includes optimizing immune status (administering effective ART), reinstating induction therapy, and susceptibility testing of isolates. If fluconazole resistance is present, salvage consolidation therapy with posaconazole, voriconazole, or high‐dose oral fluconazole (800–1200 mg a day) for 10–12 weeks can be considered [12]. In our case, the only azole antifungal available locally was fluconazole, so our patient was given a high dose of it.

## 4. Conclusion

In PLHIV, *Cryptococcal neoformans* can atypically affect organs other than the CNS, such as the bone. Fungal organisms, including *C. neoformans*, should be part of the differential diagnosis for PLHIV presenting with osteomyelitis in Sub‐Saharan Africa. Good treatment outcomes are possible with prolonged antifungal agent treatment. Future studies are suggested to evaluate the best treatment approach for *C. neoformans* infection in tissues other than the lungs or CNS.

## Author Contributions

Conceptualization: Linda A. Mandikiyana Chirimuta, Christina Rambanapasi, and David Musorowegomo. Writing–original draft: Linda A. Mandikiyana Chirimuta and Christina Rambanapasi. Writing–review and editing: Linda A. Mandikiyana Chirimuta, Christina Rambanapasi, David Musorowegomo, Gilbert Nyatanga, Augustine Mapfumo, and Eliot F. Chikati. Supervision: Eliot F. Chikati.

## Funding

The authors received no financial support for the research, authorship and publication of this article.

## Ethics Statement

This study is a case report utilizing data that have been collected for a parent study. Newlands Clinic is part of the International Epidemiologic Databases to Evaluate AIDS–Southern African Region (IeDEA‐SA). The participants whose data shall be used in this study completed an informed consent form, which allowed for the data collected during their routine care to be used for the purposes of research. This parent study has been approved by the Medical Research Council of Zimbabwe (MRCZ/A/1336). In addition, written informed consent was obtained from the patient described in this case report, allowing her clinical and demographic data and images to be used for this case report. The patient’s name and identification are not shared in the manuscript.

## Consent

Please see the Ethics Statement.

## Conflicts of Interest

The authors declare no conflicts of interest.

## Data Availability

Data sharing is not applicable to this article as no datasets were generated or analyzed during the current study.
